# Food Subsidy Effects on Host Foraging Behavior Shape Host–Macroparasite Infection Dynamics

**DOI:** 10.1002/ece3.72906

**Published:** 2026-01-23

**Authors:** Brendan B. Haile, Sarah A. Budischak, Meggan E. Craft, Clayton E. Cressler, Kristian M. Forbes, Richard J. Hall

**Affiliations:** ^1^ Odum School of Ecology University of Georgia Athens Georgia USA; ^2^ Center for the Ecology of Infectious Diseases University of Georgia Athens Georgia USA; ^3^ Department of Natural Sciences, Pitzer and Scripps Colleges Claremont California USA; ^4^ Department of Ecology, Evolution, and Behavior University of Minnesota St. Paul Minnesota USA; ^5^ School of Biological Sciences University of Nebraska Lincoln Nebraska USA; ^6^ Department of Biological Sciences University of Arkansas Fayetteville Arkansas USA; ^7^ Department of Infectious Diseases, College of Veterinary Medicine University of Georgia Athens Georgia USA

**Keywords:** anthropogenic change, disease ecology, food subsidy, helminths, model, resource

## Abstract

Anthropogenic food subsidies can have profound influences on wildlife behavior and health, including exposure to parasites. In many host–macroparasite systems, parasite exposure is tied to foraging behavior, but how different distributions of food subsidy shape macroparasite encounter and population‐level impacts is poorly understood. Here we modify a mathematical model of macroparasite transmission to explore how food subsidies could change parasite encounter rates and between‐host variation in parasite burdens, reflecting changes in host foraging and conspecific overlap. Hosts experience the highest average parasite abundance and associated reductions in population size when food subsidies increase and homogenize parasite encounter rates, for example when hosts center their home ranges on a point food source and overlap with many conspecifics. Conversely, hosts experience the lowest parasite abundance and impacts when subsidies result in lower and more heterogeneous parasite encounter rates, for example when multiple patchily distributed subsidies subdivide host populations and increase host commute times to food at the expense of time spent foraging. Even when resources affect other processes such as improving host immunity or fecundity, the overall effect of subsidies on infection is more strongly driven by changes in parasite encounter rates through altered foraging behavior. These patterns are robust to different effect sizes of resource subsidy on foraging and nonforaging parameters. Our findings demonstrate that resource‐driven shifts in host foraging behavior could play an integral role in determining infection dynamics for parasites with environmental (free‐living) infectious stages, with consequences for wildlife provisioning in recreational, conservation, and management contexts.

## Graphical abstract

Wildlife behavioral responses to different distributions of food subsidies could shape encounter and infection intensity of environmentally transmitted parasites. Using a mathematical model, we show that the way in which food subsidy changes transmission rate and variation in parasite burdens (e.g., through changes in time spent foraging and variation in conspecific overlap) determines host and parasite abundance. Moreover, these behavioral responses to food subsidy typically outweigh other effects of food subsidy on immunity and host or parasite fitness, with implications for how food provisioning is managed across landscapes.

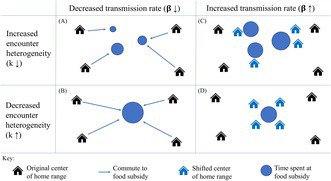

## 1 Introduction

Anthropogenic food subsidies have profound effects on wildlife physiology, behavior, and species interactions (Oro et al. 2013), including interactions with parasites (Becker, Snedden, et al. 2018; Murray et al. 2016; Strandin et al. 2018). Across diverse host taxa and parasites with different transmission modes, food subsidies that increase host population size or aggregation tend to increase transmission of microparasites (Erazo et al. 2022; Becker, Teitelbaum, et al. 2018). However, this effect does not necessarily extend to parasites with free‐living environmental infectious stages (e.g., nematodes) due to encounter‐dilution (Côté and Poulinb 1995; Patterson and Ruckstuhl 2013). However, improvements to immunity can counter these effects (Becker and Hall 2014; Forbes et al. 2016; Sweeny et al. 2021), particularly if food subsidy reduces heterogeneity in immune defense among individuals, and thus the proportion of food‐stressed superspreaders (Hall 2019). Given that human‐provided food subsidies, including agriculture, refuse, and recreational feeding, frequently bring wildlife into proximity with domestic animals and people, it is important to understand when food subsidies can lead to increased parasite transmission in wildlife and zoonotic spillover risk (Altizer et al. 2018).

The spatial scale at which food subsidies occur relative to host movement may be critical for determining parasite spread through wildlife populations. Mathematical models often assume homogeneous mixing of conspecific hosts in resource‐subsidized environments, and therefore greater food subsidy predicts increased contacts and parasite transmission (Becker and Hall 2014). However, at large spatial scales, where wildlife move infrequently among discrete resource patches, high food subsidy across the landscape can reduce parasite spread by removing resource‐based cues for dispersal (Becker, Snedden, et al. 2018; Plowright et al. 2011; Teitelbaum et al. 2020). In reality, many host interactions relevant to parasite transmission lie between fully homogeneously mixed and movement between spatially‐separated patches, and instead occur through shared space use (Wilber et al. 2022). To predict how food subsidy influences infection outcomes, it is therefore important to consider how the distribution of the food subsidy could influence host foraging behavior and conspecific overlap. A large point food source, such as a landfill, could increase host overlap and alter parasite exposure. For example, White Storks ( *Ciconia ciconia* ) in Spain nest at higher densities close to landfills (López‐García and Aguirre 2023), and birds nesting near landfills have smaller foraging ranges and preferentially use landfills (Gilbert et al. 2016); together, these potentially intensify conspecific overlap and exposure to parasites such as antibiotic‐resistant *E. coli* (Höfle et al. 2020). In contrast, more patchily‐distributed resource subsidies (e.g., agricultural mosaics) could reduce parasite transmission if hosts increase their commute times to exploit these resources (thus reducing total time spent at transmission sites), and by subdividing hosts among multiple patches, reducing conspecific overlap and parasite sharing.

Infections by macroparasites encountered during foraging, including fecal‐oral transmission by environmental infectious stages (e.g., embryonated eggs or L3 larva), are widespread, debilitating, and sometimes deadly in wildlife (Hassell et al. 2017; Petney and Andrews 1998) and livestock (Arsenopoulos et al. 2021; Kenyon et al. 2009; van Dijk et al. 2008). Macroparasite infections are characterized by inter‐individual variation in parasite infection that determines the severity of their effect on individual hosts (i.e., macroparasite induced mortality is intensity‐dependent). Distributions of parasite intensity are commonly represented using a negative binomial distribution, because macroparasites are disproportionately distributed among hosts, with some hosts having many parasites, while many hosts have few (Anderson and May 1979). While some of this variation can be explained by genetic or demographic factors (Wilson et al. 2002), part of this heterogeneity may be driven by variation in encounter with environmental parasites (Coulson et al. 2018; Peacock et al. 2020; Spiegel et al. 2022). Aggregation of hosts around food resources (Gajewski et al. 2024), behavioral avoidance of highly parasitized environments (Hutchings et al. 2000; Weinstein, Buck, and Young 2018; Weinstein, Moura, et al. 2018; Buck et al. 2018; Philippon et al. 2023), or density‐dependent departure from foraging areas with high numbers of conspecifics (Charnov 1976) may cause nonrandom mixing of hosts, further complicating parasite encounter heterogeneity. While little is known about how the distribution of food subsidy alters macroparasite infections and impacts on wildlife populations, such knowledge could be crucial for developing management interventions for wildlife using food subsidies.

Here we modify a classic model of host–macroparasite dynamics to incorporate effects of food subsidy on host behavior and physiology. Specifically, we explore how responses of host foraging behavior to different distributions of food subsidy could shape parasite encounter rate and among‐host heterogeneity in encounter. The model is applied to a hypothetical rodent parasite system where the parasite has a direct life cycle and short‐lived infectious environmental stage that is fecal‐orally transmitted (e.g., nematodes such as *Heligmosomus mixum, Heligmosomoides glareoli*, and *Aspiculuris tianjinensis*) (Grzybek et al. 2015; Behnke et al. 2015). We focused on rodents because they readily use anthropogenic food subsidies and are amenable to experimental manipulation of food and helminths (Forbes et al. 2015; Mistrick et al. 2024; Pedersen and Greives 2008). Experiments have shown that providing high‐quality food to rodents can increase clearance of helminth infection (Sweeny et al. 2021), but field sampling showed that rodents in croplands experience higher diversity and prevalence of helminth infections than populations in natural areas (Froeschke and Matthee 2014). Rodents can pose a significant health risk to humans due to their frequent occurrence in human‐altered habitats and are reservoirs of zoonotic diseases including bubonic plague, hantaviruses, and leishmaniasis (Alcover et al. 2021; Han et al. 2016; Himsworth et al. 2013; Semenza and Menne 2009; Vaheri et al. 2013). Since high helminth burdens may make rodents more susceptible or tolerant to zoonotic parasites (Hassell et al. 2017; Himsworth et al. 2013; Sweeny et al. 2020), understanding rodent‐helminth responses to food subsidy has important implications for human health.

## 2 Materials and Methods

### 2.1 Model Structure

We explored population dynamics of a single host and environmentally transmitted parasite by modifying a classic mathematical model of macroparasite transmission (Anderson and May 1979; May and Anderson 1979).

(1) $$\frac{\mathrm{dH}}{\mathrm{dt}}=\left(b-d\right)H\left(1-\frac{H}{H_{\max}}\right)-\mathrm{\alpha P}$$

(2) $$\frac{\mathrm{dP}}{\mathrm{dt}}=\mathrm{\beta HE}-\left(d+\mu \right)P-\mathrm{\alpha P}\left(1+\frac{\left(k+1\right)P}{\mathrm{kH}}\right)$$

(3) $$\frac{\mathrm{dE}}{\mathrm{dt}}=\mathrm{\lambda P}-\mathrm{\gamma E}-\beta HE$$

Under the assumption that environmental infectious stages are short‐lived relative to the adult stage (as observed in some nematodes including *H. mixum, H. glareoli*, and *A. tianjinensis*), the dynamics of free‐living stages (*E*) occur on a faster timescale than those of the host or adult parasites. Thus, a “quasi‐equilibrium approximation” can be made to obtain an approximate expression for *E* by solving Equation (3) at equilibrium and substituting this expression into Equations (1 and 2) (see Fenton (2008) for a more detailed derivation). The model then simplifies to two equations for the change in hosts (*H*) and the parasite population within hosts (*P*) through time (Anderson and May 1979; May and Anderson 1979; Deter et al. 2008; Fenton 2008; Erazo et al. 2022):

(4) $$\frac{\mathrm{dH}}{\mathrm{dt}}=\left(b-d\right)H\left(1-\frac{H}{H_{\max}}\right)-\mathrm{\alpha P}$$

(5) $$\frac{\mathrm{dP}}{\mathrm{dt}}=\frac{\text{βλPH}}{\gamma +\mathrm{\beta H}}-\left(d+\mu \right)P-\mathrm{\alpha P}\left(1+\frac{\left(k+1\right)P}{\mathrm{kH}}\right)$$

In the absence of infection, the host population exhibits logistic growth with per capita birth and death rates *b* and *d* respectively, and carrying capacity *H*_max_. Host mortality from parasite infection occurs at a rate which is the product of the per‐parasite host mortality (*α*), the average number of parasites per host (*P*/*H*), henceforth referred to as parasite abundance, and the host population size (*H*). Environmental parasite stages are shed into the environment at per parasite rate λ and lost through natural mortality (at rate γ) or ingestion by hosts (at rate *β*). The first term in the parasite equation reflects the rate of new parasite infections in hosts. The second term reflects internal parasite mortality through natural host mortality and natural or immune‐mediated parasite mortality in living hosts (at rate μ). The final term in Equations (2 and 5) represents parasite mortality due to parasite‐induced host death, with a correction term that accounts for parasite aggregation in hosts, determined by the parameter *k*. The aggregation parameter *k* takes low values when few hosts have many parasites, while large *k* reflects a roughly equal parasite abundance among hosts. A full derivation of the functional forms of these terms can be found in Anderson and May (1979) or May and Anderson (1979). Definitions of model parameters, and sources of their default values, are provided in Table 1.

**TABLE 1:** Definition of model parameters, their default values in the absence of resources set as monthly rates (when applicable), and the potential direction of the effect of resource subsidy (none, 0; increasing, +; and decreasing, −) explored in simulations.

Type	Category	Description and symbol	Value	Potential effect of resources	Potential mechanisms of resource effects
Foraging parameters $\rho_{f}$	Parasite infection via host behaviors	Infection rate, *β*	$5.2\times 10^{-8}$	0, +, −	Subsidy‐induced changes in foraging alter parasite transmission rate.
		Parasite aggregation, *k*	1.0	0, +, −	Changes in foraging alter variation in parasite abundance.
Nonforaging parameters^a^ $\rho_{\mathrm{nf}}$	Host demography	Host birth rate, *b*	0.598	0, +	Subsidy increases number of pups per litter.
		Host natural mortality, *d*	0.2	0, −	Subsidy increases host survival.
		Host carrying capacity, *H*_max_	70	0, +	Subsidy reduces intraspecific competition.
	Host immune function	Adult parasite mortality, *μ*+	0.375	0, +	Subsidy increases host clearance of parasites.
		Parasite virulence, *α*	$7.94\times 10^{-3}$	0, −	Subsidy increases host tolerance of infection.
	Parasite demography	Adult parasite mortality, *μ*−	0.375	0, −	Parasites siphon resources from hosts, increasing parasite lifespan.
		Parasite egg production, *λ*	90,000	0, +	Subsidy increases parasite egg production.
		Environmental parasite mortality, *γ*	0.5	0	Environmental parasite survival is unaffected by subsidy.

^a^ Nonforaging parameters may be altered by resource subsidy individually and in combination with foraging parameters.

### 2.2 Resource Subsidy Effects on Host and Parasite Processes

To account for effects of resource subsidy on host and parasite processes we define the resource effect size on host or parasite parameter *x*, $\rho_{x}$, as the fold change in the associated model parameter due to subsidy relative to its subsidy‐free value (Table 1):

(6) $$x=\left\{\begin{array}{c} \rho_{x}x_{0},\;\mathrm{If}\;x\;\text{increases with food subsidy}\\ \frac{x_{0}}{\rho_{x}},\;\text{If}\;x\;\text{decreases with food subsidy} \end{array}\right.$$

where $x_{0}$ is the baseline value of the parameter in the absence of food subsidy. In other words, *ρ* = 1 implies no effect of food subsidy, whereas *ρ* = 2 would double a parameter that increases with subsidy, and halve a parameter that decreases with subsidy.

We hypothesized that an individual host's foraging range, time spent foraging, and the extent to which it overlaps with other hosts influence both average parasite transmission rates (*β*) as well as individual variation in parasite encounter. We assume that time available for foraging is primarily determined by external environmental constraints, such as commute distance to the food source, and therefore that an increased proportion of time spent foraging in a host's daily activity budget increases parasite transmission (*β*). This assumption applies to animals with relatively short food handling times, such as rodents, but may not apply to species using subsidy that takes a long time to consume and digest their food. Further, we assume that resource subsidies remain attractive to foragers even at high conspecific density, as has been observed in diverse wildlife systems (Becker et al. 2015). Finally, we assume that variation in parasite transmission rates, arising from differences in time spent foraging and the intensity of space use while foraging, results in variation in the number of parasites per host as described by the aggregation parameter *k*.

### 2.3 Model Analysis

We solved the model numerically in R Statistical Software (v4.1.3; Posit Team 2022) and recorded how the host population (*H*) and average parasite abundance (i.e., total parasite population in hosts divided by host population size, *P*/*H*) changed over time in the presence and absence of food subsidy for different scenarios of resource effects on host foraging behavior, immune and demographic processes.

**FIGURE 1:**
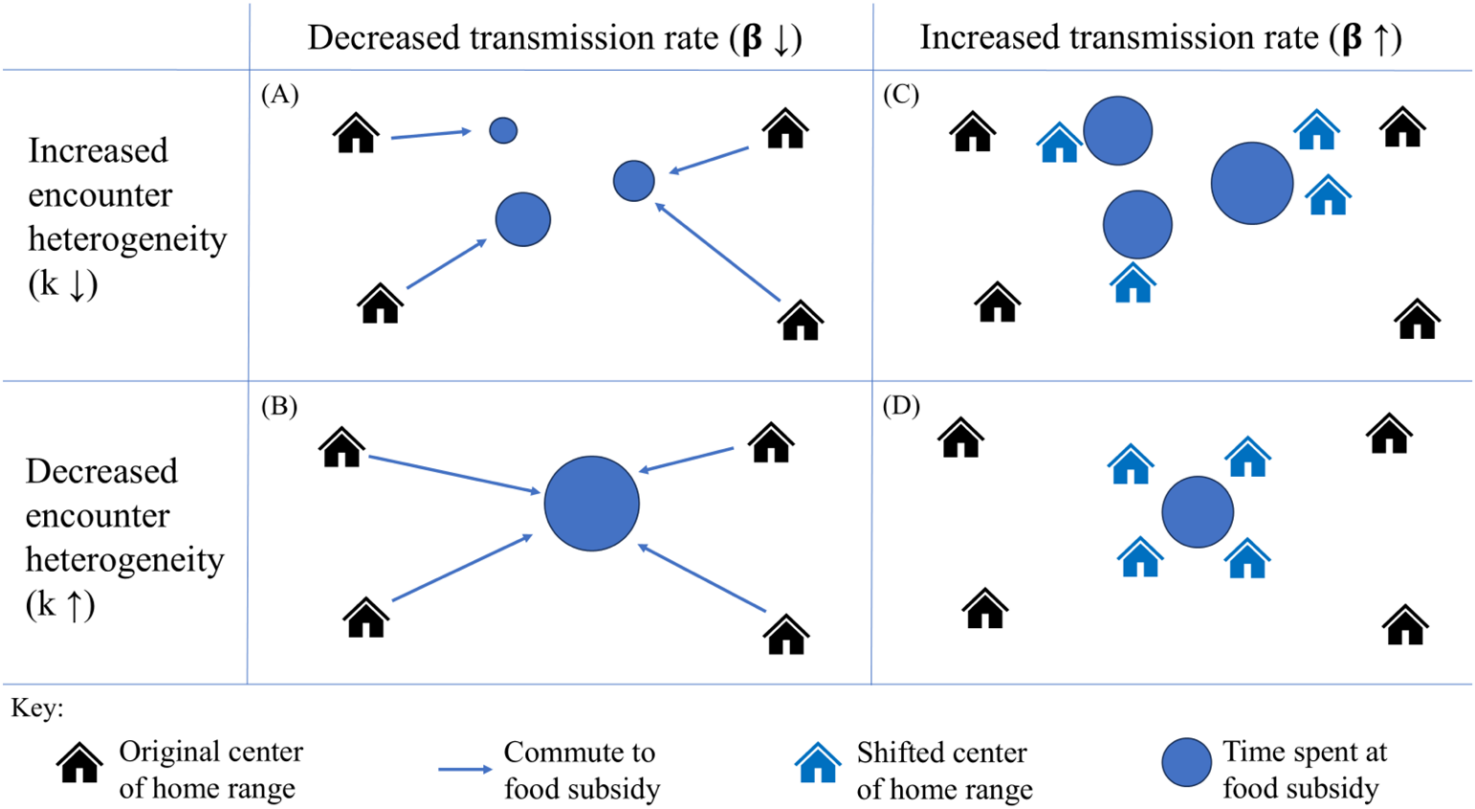
Schematic showing potential responses of host foraging to different kinds of food subsidy. Circles indicate the location of food subsidies (top row, multiple food subsidies across the landscape; bottom row, single food subsidy) and circle size indicates the time hosts spend foraging at each subsidy. Black houses indicate the centers of each host's original home range. Hosts respond to food subsidy by either commuting to food sources from their original home range, reducing time spent foraging (blue arrows) or shifting the center of their original home range closer to food subsidies (blue houses), increasing time spent foraging. Left column: Hosts commute to food subsidies, decreasing parasite transmission rate (*β*); (A) exploiting multiple food subsidies creates heterogeneity in host overlap and parasite encounter (decreasing *k*), or (B) exploiting a single food subsidy homogenizes parasite encounter. Right column: Hosts shift their ranges closer to food subsidies, increasing parasite transmission rates; with (C) distributed food subsidies increasing heterogeneity or (D) a point food source homogenizing parasite encounter.

We explored five scenarios for how food subsidy affects foraging behavior (Figure 1): a “null” scenario where food subsidy does not change foraging behavior, and four scenarios where resource subsidies cause increases or decreases in parasite transmission rate (*β*) and heterogeneity in encounter rates among individuals (and thus heterogeneity in parasite abundance, *k*). Increases in parasite transmission rate could arise when hosts shift their home ranges toward food subsidies, allowing them to increase time spent foraging (and thus ingesting parasites). Decreased parasite transmission rates could arise if hosts extend their home ranges to commute to food subsidies but maintain the core of their original home range (e.g., because they are constrained by availability of breeding or roosting sites). Increases in parasite encounter heterogeneity could arise when food subsidies are distributed across the landscape, leading to variation in spatial overlap with other hosts (and as a result, increased among‐host variation in parasite burdens). Decreases in encounter heterogeneity could arise when all hosts in a population aggregate at a point food source, reducing variation in overlap with conspecifics and homogenizing parasite infection. Our five scenarios are:

- NULL: Foraging behavior is not changed by subsidy.
- (A): A reduced parasite transmission, increased heterogeneity scenario where food subsidy reduces host exposure to parasites (reducing *β*) and increases variation in parasite encounter rate (and thus parasite abundance, reducing *k*) among hosts. This could occur when hosts commute to exploit patchily distributed resources (Figure 1A).
- (B): A reduced parasite transmission, reduced heterogeneity scenario (reduced *β*, increased *k*) where all hosts commute to a shared point food source (Figure 1B).
- (C): An increased parasite transmission, increased heterogeneity scenario (increased *β*, reduced *k*) where hosts shift their ranges toward resource subsidies, subdividing the population among discrete resource patches and increasing time available for foraging (and thus parasite transmission) (Figure 1C).
- (D): An increased parasite transmission, reduced heterogeneity scenario (increased *β* and *k*), which could occur when all hosts in the population shift their ranges toward a single food source (Figure 1D).

For each foraging scenario, one adult macroparasite was introduced to a naïve host population at its unsubsidized carrying capacity. We first explored the effects of food subsidy on foraging parameters only, and for simplicity we assumed subsidy had the same effect size on the two foraging parameters (i.e., $\rho_{\beta }=\rho_{k}=\rho_{f}$). We varied the effect of resources on both the average parasite transmission rate (*β*) and encounter heterogeneity (*k*) from none to twofold (i.e., $1<\rho_{f}<2$) and ran the model until it approached equilibrium (typically within 25 host generations or 60 months).

Next, we explored how combined effects of food subsidy on host foraging behavior and host and parasite fecundity and survival influenced host population size and parasite abundance. In these simulations, we assumed that resource subsidy resulted in a twofold change in one nonforaging parameter (e.g., host birth rate, parasite virulence, parasite egg production, etc.; See Table 1) when foraging behavior was (NULL) unaltered by food (Figure 1A), hosts commuted to distributed resources (*β* and *k* were halved) (Figure 1B), hosts commuted to a point food source (*β* halved and *k* doubled) (Figure 1C), hosts shift home range toward distributed food sources (*β* doubled and *k* halved) and (Figure 1D) hosts shift home ranges to a point food source (*β* and *k* doubled). We quantified the impacts of resource subsidy on the host population and parasite abundance by calculating the percentage change in their equilibrium values in the presence and absence of food subsidy. Thus, we define the host impact,

(7) $$\mathrm{\Delta H}=\frac{H\left(\rho_{f},\rho_{\mathrm{nf}}\right)-H\left(\emptyset \right)\;}{H\left(\emptyset \right)}\times 100$$

and the parasite impact as follows:

(8) $$\Delta \left(P/H\right)=\frac{\frac{P\left(\rho_{f},\rho_{\mathrm{nf}}\right)}{H\left(\rho_{f},\rho_{\mathrm{nf}}\right)}-\frac{P\left(\emptyset \right)}{H\left(\emptyset \right)}}{\frac{P\left(\emptyset \right)}{H\left(\emptyset \right)}}\times 100.$$

Here, we define $H\left(\emptyset \right)$ as the null scenario in which there are no effects of resource subsidy on the host, and $H\left(\rho_{f},\rho_{\mathrm{nf}}\right)$ as the equilibrium host population when food subsidy has effect size $\left(\rho_{f}\right)$ on the foraging parameters $\left(\beta ,k\right)$ and $\left(\rho_{\mathrm{nf}}\right)$ on one of the nonforaging parameters (*b*, *d*, *H*_max_, α, μ+, λ, μ−). A positive impact value indicates that the host population (or parasite abundance) increases under subsidy, and a negative value indicates a decline under resource subsidy.

In some simulations, food subsidy effects on foraging parameters only, and food subsidy effects on a nonforaging parameter only, have opposing impacts on host and parasite abundance (e.g., resource‐induced changes to foraging increase host population size while resource‐induced changes to parasite replication decrease host population size). In these cases, it is unclear what the net directional effect of resources on both nonforaging and foraging parameters will be. Therefore, we conducted a sensitivity analysis where we varied the resource effect on foraging parameters and found the corresponding value of the resource effect size on each nonforaging parameter that caused the net impact on the host or parasite to be zero (Equations 7 and 8). We plotted the resulting relationship between $\rho_{f}$ and $\rho_{n}f$, fitted a regression line and interpreted the slope value as a metric of sensitivity. Slope values greater than one indicate that resource effects on foraging drove the net direction of host and parasite population dynamics. Therefore, high slope values meant that realistically changes to nonforaging parameters were unlikely to reverse the direction of foraging effects. Additional details of this methodology are given in Appendix S1.

### 2.4 Parameterization

We parameterized the system using estimates from a previous model of a vole–nematode interaction (Deter et al. 2008); additionally, the per parasite effect on host mortality was taken from Berding et al. (1987). The default values of each parameter and hypothesized direction of food subsidy effects (Hines et al. 2007; Sweeny et al. 2020; Huang et al. 2022) are given in Table 1.

## 3 Results

### 3.1 Effects of Resource‐Induced Changes to Foraging on Infection Outcomes

**FIGURE 2:**
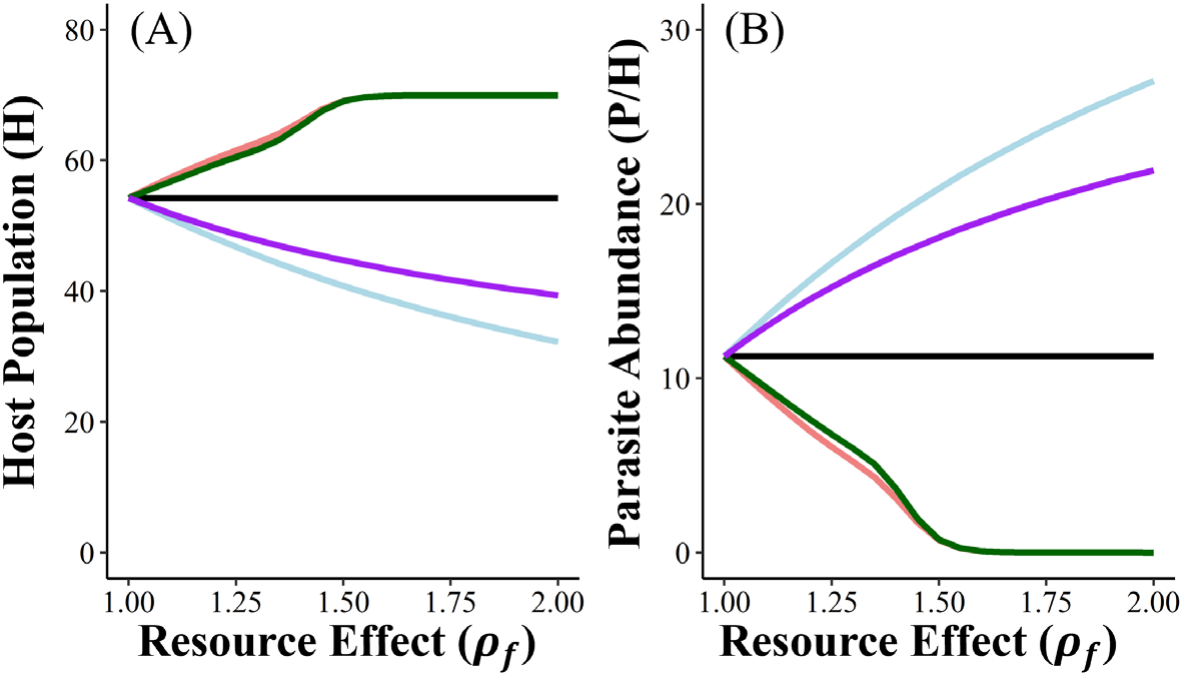
Resource‐driven changes to the parasite transmission rate determine the direction of the response of host population and parasite abundance to subsidy, while changes to parasite encounter heterogeneity dampen or exacerbate the magnitude of resource effects on these outcomes. Figures show how resources causing a fold change (of $\rho_{f}$) in both the transmission rate (*β*) and heterogeneity (*k*) influence (A) host population size (*H*) and (B) parasite abundance (i.e., the average number of parasites per host, *P*/*H*) at equilibrium. Colored lines correspond to the five scenarios for how resources affect foraging behavior: Black = no change in foraging behavior (default *β* and *k*); coral = hosts commute to resource subsidy distributed throughout environment (reducing parasite transmission rate, *β* and encounter heterogeneity, *k*); green = hosts commute to a single food subsidy (decreasing *β* and increasing *k*); blue = hosts shift home range to increase proximity to a single point food subsidy (increasing *β* and *k*), and purple = hosts shift home range to increase proximity to an environmentally distributed food subsidy (increasing *β* and reducing *k*).

We first explored how resource effects on host foraging‐related infection parameters (*β* and *k*) influenced equilibrium host population size (Figure 2A) and parasite abundance (Figure 2B). When hosts extend their foraging ranges to commute to food subsidies, the associated reduction in the parasite transmission rate (*β*) reduced overall parasite production (most hosts had very few parasites), and when resource effects were large, reduced the parasite population to the point of extinction, allowing host populations to approach their carrying capacity. The effects of food subsidy distribution via encounter heterogeneity (*k*) were relatively weak: distributed food subsidies that increased encounter heterogeneity caused slightly larger reductions in parasite abundance than a point food source that homogenized parasite encounter. In contrast, when hosts shift their home ranges toward food subsidies, spending more time foraging and ingesting parasites (i.e., increasing *β*), mean parasite abundance increased and host populations decreased. The distribution of food subsidies now had a stronger effect: point food sources that homogenize parasite encounter among hosts caused a larger increase in parasite abundance, and associated stronger regulation of host populations, than distributed food subsidies that exacerbate encounter heterogeneity. In summary, food subsidy effects on parasite transmission rate (*β*) determine the direction of change in host population dynamics and parasite abundance, whereas effects on encounter heterogeneity among hosts that influence parasite distribution (*k*) alter the magnitude of the change.

### 3.2 Combined Effects of Resources on Foraging and Nonforaging Parameters

**FIGURE 3:**
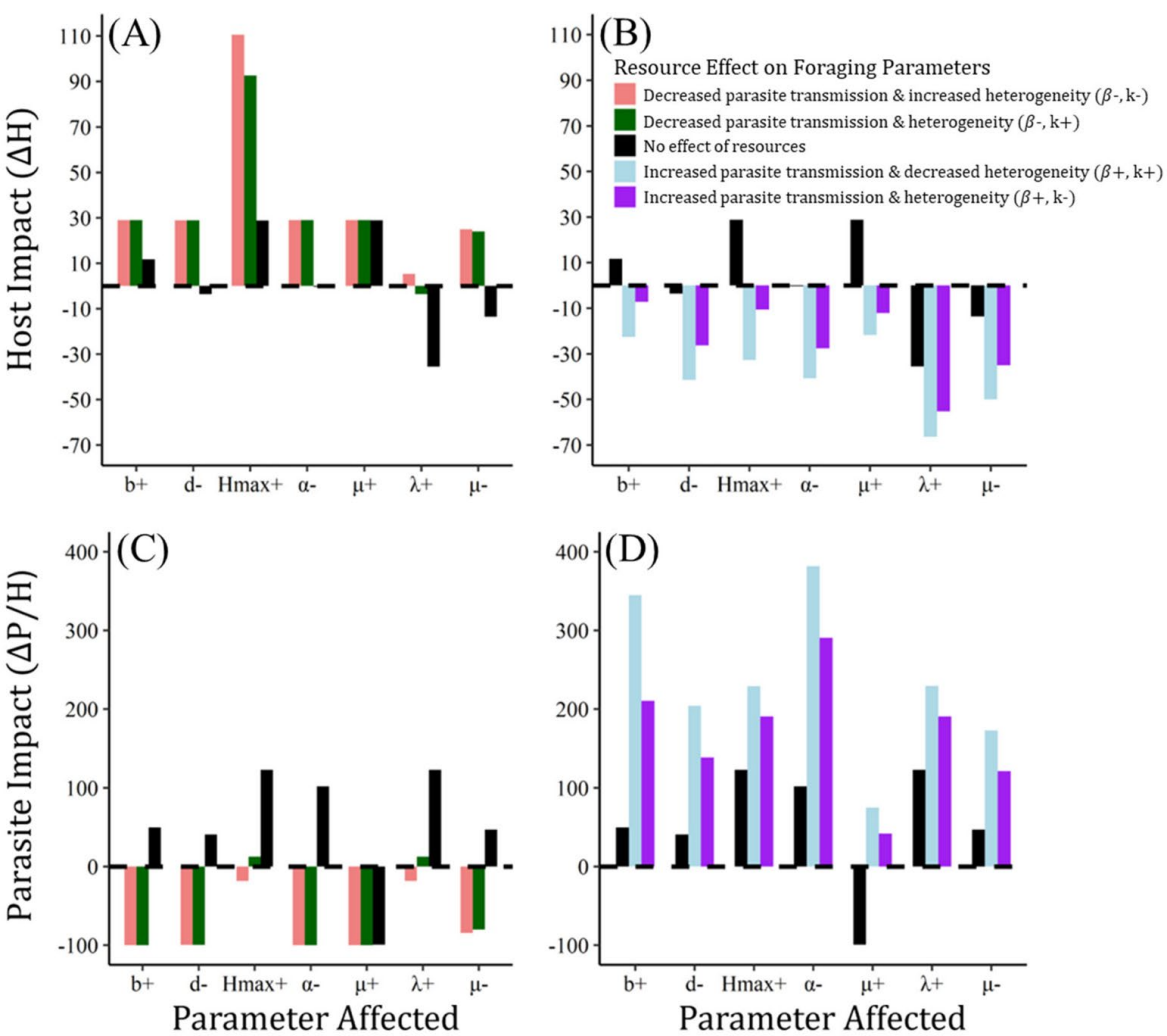
Effects of food subsidy on foraging determine the direction of change in host population or parasite abundance, even when similarly‐sized changes to host and parasite demography and immunity with subsidy have the opposite effect on host and parasite abundance. Each figure shows the impact of food subsidy on (A, B) the host population and or (C, D) parasite abundance (measured by the percent change in host or parasite abundance at equilibrium relative to an unsubsidized population), when subsidy influences one nonforaging parameter (each symbol on the horizontal axis) for each foraging scenario (bar colors). The black bars indicate a twofold change in one nonforaging parameter ($\rho_{\mathrm{nf}}=2$) when there is no effect of subsidy on foraging behavior. Each letter is the parameter affected, with + or − indicating whether the parameter increases or decreases with food subsidy, grouped by resource effects that improve population growth (increased birth rate, *b*, or carrying capacity, *H*_max_, or decreased death rate, *d*), host immune function (decreased parasite‐induced mortality rate of host, *α*, or increased parasite mortality rate, *μ*+) or parasite population growth (increased parasite egg production, or reduced parasite death rate, *μ*−). Other colored bars indicate a two‐fold change in the nonforaging parameter and the foraging parameters (transmission rate, *β* and encounter heterogeneity, *k*) for each foraging scenario (see inset legend). The dashed horizontal line denotes no net effect of food subsidy; bars above this line indicate a net positive impact of subsidy (i.e., increased host or parasite abundance), and bars below the line indicate a net negative impact of subsidy.

When resource subsidies affected one nonforaging parameter only and did not change host foraging behavior, the magnitude and direction of the effect of resources on host populations and parasite abundance varied according to the host or parasite process affected (Figure 3). In most scenarios, resource subsidies increased parasite abundance; resource effects that doubled host carrying capacity, halved parasite‐induced host mortality, or doubled parasite production had the strongest influence (roughly doubling parasite abundance). The only resource effect that reduced parasite abundance occurred when improved host immune defense increased parasite mortality within the host. The response of host population size was more variable in its direction and generally weaker in its magnitude (±30% change from baseline) than effects on parasite abundance. Host populations mostly covaried negatively with parasite abundance (reflecting parasite regulation of the host), but when resource subsidies increased host birth rate or carrying capacity, parasite abundance and host population size both increased. The strongest positive effects on host population size occurred when resources increased host carrying capacity or immune‐mediated parasite mortality, and the strongest negative effect was observed when resources increased parasite egg production.

When hosts extended their foraging ranges to commute to resource subsidies (decreasing *β*), and subsidy influenced one nonforaging parameter, almost all parameter combinations resulted in larger host population sizes and lower parasite abundance compared to the subsidy‐free baseline (Figure 3A,C). The only exception was when a point food subsidy homogenized parasite encounter among hosts (increasing *k*) and subsidy increased parasite egg production (λ), which counteracted the reduction in parasite uptake and resulted in parasite abundance and host population size similar to an unsubsidized population. When resource subsidies increased parasite transmission, host populations always decreased and parasite abundance increased irrespective of the resource effect on nonforaging parameters (Figure 3B,D). Together these suggest that effects of food subsidy on host foraging behavior generally override counteracting effects of food on host and parasite vital rates. These effects are largely driven by how host foraging rates (and thus parasite transmission, *β*) change with food subsidy, while changes in encounter heterogeneity (and thus parasite aggregation, *k*) have a moderating effect that either exacerbates or lessens the effects of subsidy on parasite transmission.

### 3.3 Sensitivity Analysis

**FIGURE 4:**
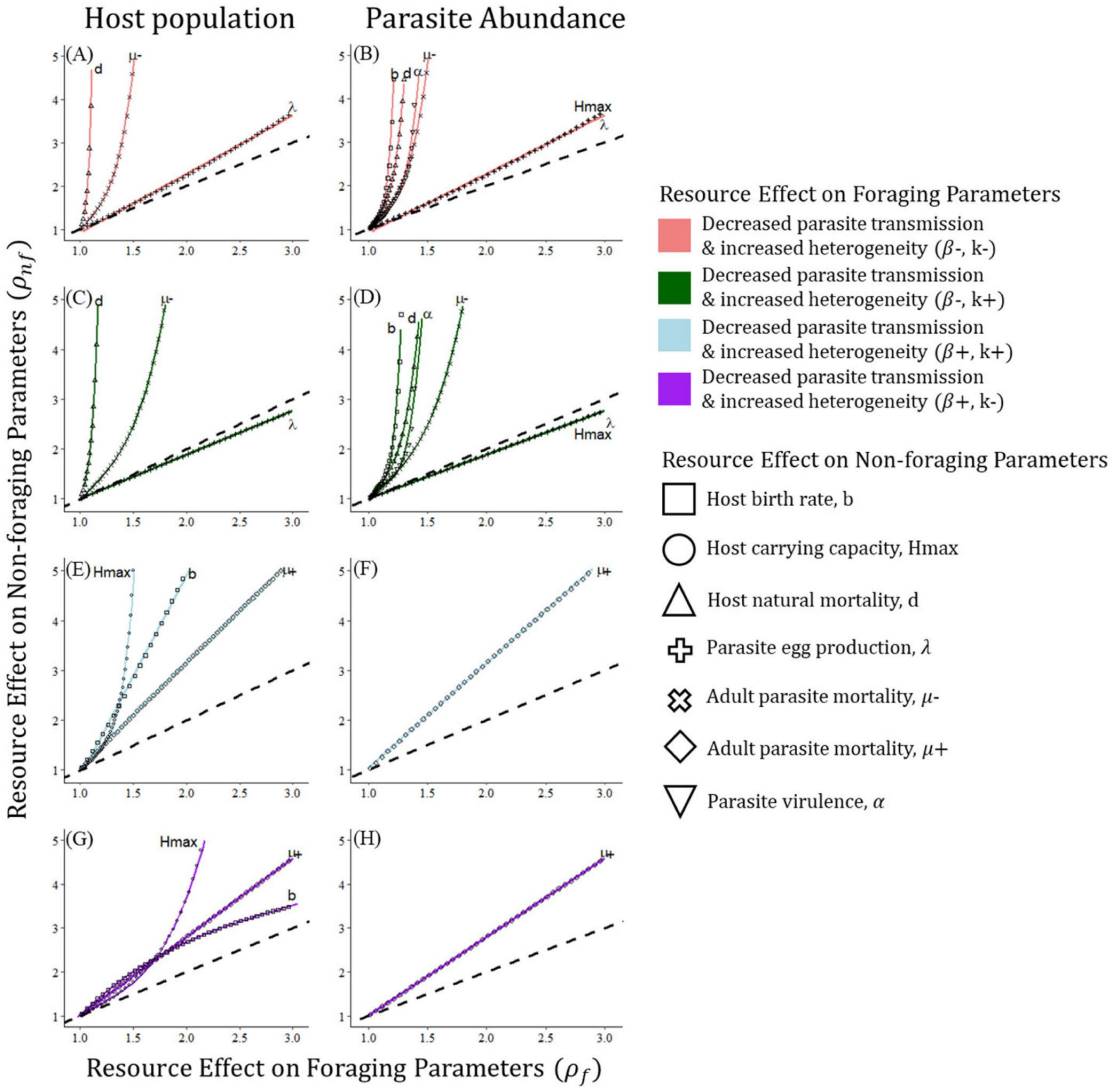
Resource effects on demographic and immune parameters generally have to be stronger than resource effects on foraging to influence the net direction of change in host and parasite abundance in response to food subsidy. Sub‐figures show the effect of resources on a nonforaging parameter ($\rho_{\mathrm{nf}}$, vertical axis) required to overcome the effect of resource subsidies on foraging ($\rho_{f}$, horizontal axis) and change the sign of the resource impacts on host population (left column) and parasite abundance (right column). Symbols represent simulated model outputs for each nonforaging parameter with fitted regression lines overlaid. Rows and line colors correspond to the four scenarios through which resources alter foraging: (A, B) resources decrease parasite transmission and increase encounter heterogeneity (*β*−, *k*−, coral); (C, D) resources decrease parasite transmission and heterogeneity (*β*−, *k*+, green); (E, F): Resources increase transmission and decrease heterogeneity (*β*+, *k*+, blue), and (G, H) resources increase transmission and heterogeneity (*β*+, *k*−, purple). The dashed line represents the one‐to‐one line where overturning the effects of a fold change in foraging parameters requires an equal fold change in a nonforaging parameter; the further the line for each nonforaging parameter is above the one‐to‐one line, the less likely it is that nonforaging effects of resource subsidy can overturn resource effects on foraging. Symbol type indicates nonforaging parameter affected by food subsidy: *b* (host birth rate, □), *H*_max_ (host carrying capacity, ○), *d* (host natural mortality rate, △), *λ* (parasite egg production, +), *μ*− (adult parasite mortality rate, ✖), *μ*+ (adult parasite mortality rate, ◇), *α* (parasite virulence, ▽).

When resources affected foraging and nonforaging parameters equally, we found that parasite transmission (*β*) typically determined the net direction of the change in population size and parasite abundance, while parasite encounter heterogeneity among hosts (*k*) modified the magnitude but not direction of the change (Figure 3). To further assess the generality of this finding, for each nonforaging parameter, we plotted the resource effect size ($\rho_{\mathrm{nf}}$) needed to overturn the effects of resources on foraging for host population size and parasite abundance (Figure 4, Table A1). When resource subsidy reduced parasite transmission (*β*), resource‐elevated parasite egg production (λ) was the most likely nonforaging parameter to be able to overturn the beneficial effects of reduced parasite transmission and lead to increased parasite abundance and decreased host population size. Additionally, resource‐driven increases in host's carrying capacity (*H*_max_) could counteract effects of reduced transmission to increase parasite abundance, but not host population size (Figure 4A–D). These patterns held whether resources increased or decreased parasite encounter heterogeneity (*k*), but it was easier for resource effects on foraging to be overturned when resources homogenized parasite encounter (Figure 4C vs. Figure 4A, Figure 4D vs. Figure 4B). When resource subsidy increased parasite transmission, resource increases to host immune response (via elevated internal parasite mortality, 𝜇+) and host birth rate (*b*) could overturn reductions in host population size due to increased exposure (Figure 4E,G); however, only resource‐improved immunity could reduce parasite abundance (Figure 4F,H). Foraging effects were more easily overturned by these nonforaging parameters when resources increased heterogeneity in parasite encounter. For all scenarios, the effects of resources on all other nonforaging parameters required to overturn effects on foraging increased faster than linearly with resource effects of foraging, meaning it was unlikely they could ever overturn foraging effects in biologically plausible scenarios.

## 4 Discussion

Responses of host foraging behavior to different distributions of food subsidy, and their consequences for transmission of parasites acquired environmentally, are poorly understood. Using a host–macroparasite model that incorporated mechanistic hypotheses for how resource subsidies alter host foraging behaviors relevant to parasite transmission and encounter, we found that behavioral responses to different resource distributions had opposing outcomes for hosts and parasites. When hosts relocated their home range closer to resource subsidies, increasing time spent ingesting parasites while foraging, they experienced higher parasite abundance and consequently reduced population size. In contrast, hosts that commuted to resource subsidies (reducing time spent foraging and ingesting parasites) experienced lower parasite abundance and increased population size. Effects of resource subsidy on parasite encounter heterogeneity (arising from different distributions of subsidies across landscapes) modified the strength, but not the direction, of food subsidy effects on host population and parasite abundance. Strikingly, the effects of food subsidy distribution on host foraging behavior almost always outweighed the beneficial effects of food subsidy on other demographic and infection processes. These resource subsidy‐induced changes to foraging and infection may scale up to influence landscape‐level patterns of disease spread. From an applied perspective, our findings suggest that wildlife managers and recreational wildlife feeders could mitigate effects of food subsidy on parasite transmission by distributing provided food to prevent aggregation of animals and environmental parasite stages.

Our model simulations suggest that animals relocating their home ranges to exploit a point food subsidy experience the largest detrimental impact on their population size because it promotes resampling of areas where host aggregations lead to accumulation of environmentally persistent parasites. Findings from several empirical studies are consistent with our model predictions. White Storks in Europe have been found to nest in close proximity to landfills, allowing large nonmigratory populations to persist despite the repercussions of increased parasitism (Gilbert et al. 2016; Höfle et al. 2020; López‐García and Aguirre 2023). Populations of raccoons, elk, and wild boar provided with point food sources for conservation, game management, or research purposes reduced their home ranges and experienced increased conspecific aggregation, consequently increasing parasite encounter due to shared space usage (Gompper and Wright 2005; Hines et al. 2007; Navarro‐Gonzalez et al. 2013).

In agreement with our model results, we found empirical studies that explored the effect of spatially‐distributed food resources on host behavior and space sharing showed reductions in parasite transmission. Gompper and Wright (2005) found that raccoons receiving resource subsidies that were distributed throughout the landscape remained solitary and had significantly fewer contacts with conspecifics. The impact of distributed food subsidy varies in rodent populations, with highly nutritious supplements leading to a significant decrease in parasite abundance and egg production for both lab and field experiments of *Apodemus sylvaticus* (Sweeny et al. 2021). A recent study that manipulated resource availability in wild bank voles ( *Clethrionomys glareolus* ) found mixed effects on individual space use and conspecific overlap that varied by sex, season, and reproductive status (Mistrick et al. 2024), suggesting that seasonal and demographic factors may be important considerations for how resources shape parasite exposure through foraging.

### 4.1 Confounding Factors and Future Research Directions

We created a simple mathematical model to create testable hypotheses on the implications of food subsidy and the underlying importance of foraging behaviors in a single host–macroparasite system. However, we believe that the model may provide general insight for many systems, particularly when modified to incorporate system specific processes (e.g., optimal foraging theory, multi‐host–parasite systems, or coinfection). Species with behavioral adaptations to limit or avoid parasite encounter, including foraging strategy (grazing vs. browsing or dietary shifts), use of middens/latrines, avoidance of detectable odors in urine of infected individuals, and migration (Gompper and Wright 2005; Kavaliers and Choleris 2018; Majewska et al. 2019; Milner et al. 2013) may be able to exploit aggregated food subsidies while minimizing an increased risk of infection. For example, moose that visited feeding stations did not experience increased parasitism from fecal–orally transmitted parasites (Milner et al. 2013), perhaps because their browsing foraging strategy means they are choosing food less likely to be contaminated with fecal matter. However, these behavioral adaptations may be less effective under reliable food subsidy. For example, while latrine usage by solitary raccoons reduces parasite encounter, increased densities of conspecifics caused by a point food source may result in communal latrines which could promote parasite encounter (Gompper and Wright 2005). Many animals including ungulates and monarch butterflies ( *Danaus plexippus* ) migrate away from sites with high parasite pressure (Bartel et al. 2011; Gunn and Irvine 2003), but this benefit could be eroded if food subsidies cause some migrants to become residents (Brown and Hall 2018). For example, monarchs in the southeastern United States form sedentary populations in response to planted tropical milkweed, which promotes year‐round breeding and high levels of parasitism by a protozoan shed by adult butterflies visiting milkweed plants and consumed by their larvae (Majewska et al. 2019; Satterfield et al. 2015).

Since food subsidies can attract multiple species, host avoidance of predators and resource competitors could alter patterns of space use and conspecific overlap that influence transmission of specialist parasites (Barnard et al. 2003; Becker et al. 2015; Gunn and Irvine 2003). For multi‐host parasites, food subsidy could result in amplification or dilution of transmission, dependent on species‐specific competence for infection, as well as differences in abundance and space use among host species (Becker et al. 2015; Dobson 2004; Manzoli et al. 2021). Finally, hosts are typically infected by multiple coinfecting parasites that compete for host resources and modify the expression of immune defense (Budischak et al. 2012; Mabbott 2018). In addition to direct effects of food subsidy on within host processes, food subsidies could alter parasite community composition within hosts by reducing exposure to some parasites through reductions in foraging range size, changes in conspecific overlap or dietary shifts (Lane et al. 2011; López‐García and Aguirre 2023). Together, these suggest that accounting for host behavioral change and the distribution of resource subsidies could yield important insights into studies of parasite transmission in a wider community context.

While our model explores mechanisms through which changes to host foraging might result from differing resource distributions, space is not modeled explicitly in our framework. Other work exploring landscape‐level effects of food subsidy on infection dynamics suggests that food subsidy increases local parasite transmission but inhibits parasite spread across the landscape because animals at food‐subsidized sites show higher site fidelity (Becker, Snedden, et al. 2018; Plowright et al. 2011; Teitelbaum et al. 2015). This is consistent with our scenarios of home range shifts toward point or distributed food sources that increase parasite abundance per host at a site, but also suggests that reduced landscape‐level connectivity could reduce total parasite abundance at the landscape level.

We assume transmission increases with increasing time available for foraging (e.g., due to proximity of a food subsidy), but host handling times, responses to conspecific density, and negative impacts of parasite infection on foraging and inter‐site movement, could alter the nature of this relationship. Hosts with high handling time may spend less time foraging, that could result in a saturating relationship between time available for foraging and parasite acquisition. While evidence suggests that food subsidies generally increase numbers and aggregation at feeding sites (Becker et al. 2015), crowding or conspecific aggression that reduce per capita resource availability could cause individuals to depart a subsidized site, consistent with the Marginal Value Theorem (Charnov 1976). This would potentially reduce parasite exposure when a single point source of resources attracts large numbers of individuals, but for distributed resource subsidies this could increase movement of animals between sites that potentially homogenizes their parasite encounter rates. This density effect could be explored by making our foraging‐related transmission parameters (β and *k*) functions of conspecific host density.

In our model, we hypothesize that heterogeneity in host overlap leads to heterogeneity in parasite abundance; however, high parasite burdens could also reduce movement propensity in ways that influence host overlap and transmission (Peacock et al. 2020). These feedbacks between longer‐distance host movements and parasite burdens could be explored in future work that explicitly incorporates movement rules between resource unsubsidized and subsidized patches.

### 4.2 Implications for Management

Given the popularity of recreational wildlife feeding, and the widespread use of food subsidies as a conservation tool, evidence‐based guidelines that promote the benefits of feeding while reducing infection risk are urgently needed (Dayer et al. 2024; Murray et al. 2016; Roden‐Reynolds et al. 2022; Vicente et al. 2006). Our simple model provided a testable prediction that changes in foraging behavior due to subsidy were at least as impactful as changes to host or parasite fitness. While management in any focal system should be guided by models incorporating more biological realism, our study does suggest that interventions focused on reducing overall time at food subsidies (e.g., by periodically removing feeders or discouraging wildlife from breeding at subsidized sites), or breaking the connection between foraging and parasite ingestion (e.g., through sanitation of feeding areas or providing elevated feeding platforms that separate food from feces) could be beneficial (Navarro‐Gonzalez et al. 2013).

## Author Contributions

**Brendan B. Haile:** conceptualization (lead), data curation (lead), formal analysis (lead), funding acquisition (supporting), investigation (lead), methodology (lead), project administration (equal), resources (equal), software (lead), validation (lead), visualization (lead), writing – original draft (lead), writing – review and editing (lead). **Sarah A. Budischak:** funding acquisition (equal), project administration (equal), supervision (equal), writing – review and editing (equal). **Meggan E. Craft:** funding acquisition (equal), project administration (equal), supervision (equal), writing – review and editing (equal). **Clayton E. Cressler:** funding acquisition (equal), project administration (equal), supervision (equal), writing – review and editing (equal). **Kristian M. Forbes:** funding acquisition (lead), project administration (equal), supervision (equal), writing – review and editing (equal). **Richard J. Hall:** conceptualization (equal), data curation (equal), formal analysis (equal), funding acquisition (equal), investigation (equal), methodology (equal), project administration (equal), resources (equal), software (equal), supervision (equal), validation (equal), visualization (equal), writing – original draft (equal), writing – review and editing (equal).

## Funding

This work was supported by the National Science Foundation (DEB‐1911925, DEB‐2153924, DEB‐2321358, DGE‐1545433).

## Conflicts of Interest

The authors declare no conflicts of interest.

## Supporting information


**Appendix S1:** ece372906‐sup‐0001‐AppendixS1.docx.

## Data Availability

Data sets utilized for this research are as follows: Berding et al. (1987) (https://doi.org/10.1016/S0022‐5193(87)80227‐8) and Deter et al. (2008) (https://doi.org/10.1007/s10344‐007‐0110‐6). This manuscript uses novel code, which is provided for the peer review process in an external repository (https://doi.org/10.5281/zenodo.15098462).
