# Supplementary material for: Food Subsidy Effects on Host Foraging Behavior Shape Host–Macroparasite Infection Dynamics
Source: Ecol Evol. 2026 Jan 23;16(1):e72906. doi: 10.1002/ece3.72906 (PMC12831021; doi:10.1002/ece3.72906)
Supplement: Supplementary file 1 — Appendix S1: ece372906‐sup‐0001‐AppendixS1.docx. [file ECE3-16-e72906-s001.docx]

**Appendix**

**Sensitivity analysis**

In cases where food subsidy effects on foraging and non-foraging parameters had opposing effects on population and infection outcomes ($\Delta H$ and $\Delta P/H$) (e.g. when food effects on foraging parameters alone increased host population size but the effect of food on the non-foraging parameter alone reduced the population), we conducted a sensitivity analysis to determine which of these opposing effects determined the net direction of infection outcomes when food subsidy altered both. To do this, we numerically calculated the effect size of resources each non-foraging parameter $(\rho*_{nf})$ needed to reverse the direction of resource-induced changes to foraging on host population size or parasite abundance.

Here, we define $H(\emptyset)$ as the null scenario in which there are no effects of resource subsidy. We define $H(\rho_{f})$ as the equilibrium host population when food subsidy has effect size $(\rho_{f})$ on the foraging parameters ($\beta$, k). We define $H(\rho_{nf})$ as the equilibrium host population when food subsidy has effect size $(\rho_{nf})$ on one of the non-foraging parameters (b, d, Hmax, $\alpha$, $\mu$+, $\lambda$, $\mu$-). Finally, we define $H(\rho_{f},\rho_{nf})$ as the equilibrium host population when food subsidy has effect size ${(\rho}_{f})$ on the foraging parameters ($\beta$, k) and $(\rho_{nf})$ on one of the non-foraging parameters (b, d, Hmax, $\alpha$, $\mu$+, $\lambda$, $\mu$-).

If $\Delta H(\rho_{nf}) < 0 < \Delta H(\rho_{f})$ or $H(\rho_{f}) < 0 < \Delta H(\rho_{nf})$ $\Rightarrow\forall H(\rho_{f},\rho_{nf}) \exists\rho_{nf} : \Delta H(\rho_{f},\rho_{nf})+\Delta H(\rho_{f}) = \Delta H(\emptyset) = 0$.

The same mathematical expressions may be applied to determine resource effect size combinations of foraging and non-foraging parameter that result in no change in parasite impact ($\Delta P/H$). We varied the effect size of resource subsidy on foraging parameters from no effect to a three-fold change ($1<\rho_{f}<3$), and recorded the fold change in the non-foraging parameter (${1<\rho}_{nf}<5$) that nullified the net effect on host population size and parasite abundance (i.e. the food-subsidized host population size and parasite abundance were the same as those for an unsubsidized population), which we denote as $(\rho*_{nf})$. We used nonlinear least squares to fit the relationship between ${(\rho}_{f})$ and $(\rho*_{nf})$ for each parameter and used AIC to select the most parsimonious model (see Table A1).

Table A1: Resource breakpoint values and regression model outputs of host population (H) and parasite abundance (P/H) for all combinations of resource-altered foraging ($\rho_{f}$) and non-foraging parameters ($\rho_{nf}$).

| State Variable | Non-foraging Parameter | Foraging Parameters | Maximal Resource Breakpoint Values  ${(\rho}_{f} ,\rho_{nf})$ | Regression Information |
| --- | --- | --- | --- | --- |
| Host Population (H) | Host birth rate, b | Parasite encounter, $\beta+$ Distribution heterogeneity, $k+$ | (1.9875, 4.95) | $F\left( 1,77 \right)= 2.2*10^{-16}$  $R^{2}=0.9982$ |
|  | Host birth rate, b | Parasite encounter, $\beta+$ Distribution heterogeneity, $k-$ | (3.0, 3.5125) | $F\left( 1,156 \right)= 2.2*10^{-16}R^{2}=0.9999$ |
|  | Host natural mortality, d | Parasite encounter, $\beta-$Distribution heterogeneity, $k-$ | (1.10, 3.85) | $F\left( 3,4 \right)= 1.26*10^{-6}R^{2}=0.9986$ |
|  | Host natural mortality, d | Parasite encounter, $\beta-$ Distribution heterogeneity, $k+$ | (1.15, 4.10) | $F\left( 3,8 \right)=1.06*10^{-12}R^{2}=0.9989$ |
|  | Host carrying capacity, Hmax | Parasite encounter, $\beta+$ Distribution heterogeneity, $k+$ | (1.50, 5.0) | $F\left( 3,36 \right)= 2.2*10^{-16}R^{2}=0.9938$ |
|  | Host carrying capacity, Hmax | Parasite encounter, $\beta+$ Distribution heterogeneity, $k-$ | (2.15, 4.90) | $F\left( 3,88 \right)=2.2*10^{-16}R^{2}=0.9995$ |
|  | Parasite virulence, α | Parasite encounter, $\beta+$ Distribution heterogeneity, $k+$ | N/a | N/a |
|  | Parasite virulence, α | Parasite encounter, $\beta+$ Distribution heterogeneity, $k-$ | N/a | N/a |
|  | Adult parasite mortality, µ+ | Parasite encounter, $\beta+$ Distribution heterogeneity, $k+$ | (2.8875, 5.0) | $F\left( 1,149 \right)=2.2*10^{-16}R^{2}=0.9999$ |
|  | Adult parasite mortality, µ+ | Parasite encounter, $\beta+$ Distribution heterogeneity, $k-$ | (3.0, 4.5875) | $F\left( 1,158 \right)=2.2*10^{-16}R^{2}=0.9999$ |
|  | Parasite egg production, λ | Parasite encounter, $\beta-$Distribution heterogeneity, $k-$ | (3.0, 3.7125) | $F\left( 1,158 \right)=2.2*10^{-16}R^{2}=0.9979$ |
|  | Parasite egg production, λ | Parasite encounter, $\beta-$ Distribution heterogeneity, $k+$ | (3.0, 2.775) | $F\left( 1,158 \right)=2.2*10^{-16}R^{2}=0.9999$ |
|  | Adult parasite mortality, µ- | Parasite encounter, $\beta-$Distribution heterogeneity, $k-$ | (1.50, 4.9125) | $F\left( 3,60 \right)=2.2*10^{-16}R^{2}=0.9997$ |
|  | Adult parasite mortality, µ- | Parasite encounter, $\beta-$ Distribution heterogeneity, $k+$ | (1.80, 4.95) | $F\left( 3,36 \right)=2.2*10^{-16}R^{2}=0.999$ |
| Parasite Abundance (P/H) | Host birth rate, b | Parasite encounter, $\beta-$Distribution heterogeneity, $k-$ | (1.2125, 4.45) | $F\left( 3,13 \right)=8.82*10^{-14}R^{2}=0.9898$ |
|  | Host birth rate, b | Parasite encounter, $\beta-$ Distribution heterogeneity, $k+$ | (1.275, 4.70) | $F\left( 3,18 \right)=2.2*10^{-16}R^{2}=0.9851$ |
|  | Host natural mortality, d | Parasite encounter, $\beta-$Distribution heterogeneity, $k-$ | (1.30, 4.45) | $F\left( 3,20 \right)=2.2*10^{-16}R^{2}=0.998$ |
|  | Host natural mortality, d | Parasite encounter, $\beta-$ Distribution heterogeneity, $k+$ | (1.425, 4.625) | $F\left( 3,30 \right)=2.2*10^{-16}R^{2}=0.9986$ |
|  | Host carrying capacity, Hmax | Parasite encounter, $\beta-$Distribution heterogeneity, $k-$ | (3.0, 3.7125) | $F\left( 1,158 \right)=2.2*10^{-16}R^{2}=0.9979$ |
|  | Host carrying capacity, Hmax | Parasite encounter, $\beta-$ Distribution heterogeneity, $k+$ | (3.0, 2.775) | $F\left( 1,158 \right)=2.2*10^{-16}R^{2}=0.9999$ |
|  | Parasite virulence, α | Parasite encounter, $\beta-$Distribution heterogeneity, $k-$ | (1.3875, 3.875) | $F\left( 3,27 \right)=2.2*10^{-16}R^{2}=0.9743$ |
|  | Parasite virulence, α | Parasite encounter, $\beta-$ Distribution heterogeneity, $k+$ | (1.40, 4.25) | $F\left( 3,28 \right)=2.2*10^{-16}R^{2}=0.9288$ |
|  | Adult parasite mortality, µ+ | Parasite encounter, $\beta+$ Distribution heterogeneity, $k+$ | (2.8875, 5.0) | $F\left( 1,149 \right)=2.2*10^{-16}R^{2}=0.9999$ |
|  | Adult parasite mortality, µ+ | Parasite encounter, $\beta+$ Distribution heterogeneity, $k-$ | (3.0, 4.5875) | $F\left( 1,158 \right)=2.2*10^{-16}R^{2}=0.9999$ |
|  | Parasite egg production, λ | Parasite encounter, $\beta-$Distribution heterogeneity, $k-$ | (3.0, 3.7125) | $F\left( 1,158 \right)= 2.2*10^{-16}R^{2}=0.9979$ |
|  | Parasite egg production, λ | Parasite encounter, $\beta-$ Distribution heterogeneity, $k+$ | (3.0, 2.775) | $F\left( 1,158 \right)=2.2*10^{-16}R^{2}=0.9999$ |
|  | Adult parasite mortality, µ- | Parasite encounter, $\beta-$Distribution heterogeneity, $k-$ | (1.50, 4.9125) | $F\left( 3,36 \right)=2.2*10^{-16}R^{2}=0.999$ |
|  | Adult parasite mortality, µ- | Parasite encounter, $\beta-$ Distribution heterogeneity, $k+$ | (1.80, 4.925) | $F\left( 3,60 \right)=2.2*10^{-16}R^{2}=0.9997$ |
